# Role of Combined Ortho-Plastic Approaches in the Reconstruction of Gustilo–Anderson Grade III Upper Limb Injuries: A Systematic Review

**DOI:** 10.7759/cureus.93314

**Published:** 2025-09-26

**Authors:** Kshitij Srivastava, Abdelrahman Ibrahim, Miqdad Qandeel, Mirza Arsalan Baig, Abdullah Elrefae, Rewant Singh, Rao Junaid Saleem, Safeer Ahmad Javid, Muddasir Reyaz Hassan, Shahzad Ahmad

**Affiliations:** 1 Trauma and Orthopaedics, Northwick Park Hospital, London, GBR; 2 Trauma and Orthopaedics, University Hospitals of North Midlands NHS Trust, Stoke on Tent, GBR; 3 Trauma and Orthopaedics, Central Middlesex Hospital, London, GBR; 4 Trauma and Orthopaedics, London Northwest University Healthcare NHS Trust, London, GBR; 5 Trauma and Orthopaedics, AlBashir Hospital, Amman, JOR; 6 Trauma and Orthopaedics, Northwick Park Hospital, London, London, GBR; 7 General and Colorectal Surgery, Northwick Park Hospital, London, GBR; 8 Trauma and Orthopaedics, Royal Sussex County Hospital, Brighton, GBR; 9 Surgery, Liaquat National Hospital, Karachi, PAK

**Keywords:** gustilo-anderson grade iii, open fractures, ortho-plastic surgery, soft tissue coverage, upper limb reconstruction

## Abstract

This systematic review investigates the role of combined ortho-plastic approaches in the reconstruction of Gustilo-Anderson Grade III upper limb fractures, which are characterized by severe soft tissue damage, contamination, and high rates of complications such as infection and nonunion. A total of five studies, encompassing 196 patients, were analyzed following a systematic search across PubMed, Embase, Scopus, and the Cochrane Library in accordance with PRISMA 2020 guidelines. The included studies evaluated early multidisciplinary interventions involving orthopedic fixation and plastic surgical soft tissue reconstruction. Early intervention, particularly within 72 hours was associated with improved outcomes, including limb salvage rates up to 100%, reduced deep infection rates as low as 2.4%, and union rates up to 84.3%. Both single-stage and staged procedures showed positive results, with vascularized flap coverage playing a crucial role in maintaining tissue viability and promoting bone healing. The findings strongly support the use of coordinated ortho-plastic strategies to manage complex upper limb trauma, although further prospective, high-quality trials are needed to standardize timing and techniques.

## Introduction and background

A fracture occurs when the natural continuity of a bone is disrupted due to external trauma, pathological weakening, or repetitive mechanical stress. The severity of a fracture can range from a simple linear crack to complex comminuted patterns involving multiple displaced fragments. Fractures of the long bones of the upper limb, including the humerus, radius, and ulna, are particularly significant due to the complex anatomy and essential functional role of the upper extremity in performing daily activities [1]. A large-scale study reported a total of 4,890 long bone fractures, with an overall incidence of 406 per 100,000 people per year. Of these, upper limb fractures accounted for 159 per 100,000, and open fractures represented approximately 3% of all cases. While less common, open fractures are associated with higher morbidity due to soft tissue disruption, increased risk of infection, and the need for multidisciplinary management. Pediatric patients are typically managed conservatively, while more than half of adult patients require operative fixation. The use of angular stable plates has increased over time, reflecting evolving surgical strategies [2].

Open fractures of the upper extremity are commonly classified using the Gustilo-Anderson system, which is based on the degree of soft tissue damage, level of contamination, and presence of vascular injury. The classification includes Type I (clean wounds <1 cm), Type II (wounds >1 cm without extensive soft tissue loss), and Type III, which encompasses high-energy injuries with extensive soft tissue damage. Type III fractures are further subdivided into IIIA (adequate soft tissue coverage), IIIB (significant soft tissue loss requiring flap coverage), and IIIC (associated with arterial injury requiring repair) [3]. This classification assists clinicians in treatment planning and provides insight into potential complications and prognosis. Gustilo-Anderson Grade III injuries of the upper limb are among the most complex musculoskeletal traumas encountered. They involve extensive soft tissue destruction, contamination, bone exposure, and sometimes neurovascular compromise. These injuries carry a high risk of infection, non-union, and functional loss, and they frequently require surgical expertise from multiple disciplines [4-5]. The management of such injuries poses a dual challenge: achieving stable fracture fixation and providing effective soft tissue reconstruction. Historically, the orthopedic focus on bone fixation often delayed wound coverage, which was associated with increased rates of infection and poor functional outcomes [6].

Over the past two decades, there has been a paradigm shift toward a combined ortho-plastic approach. This strategy involves close collaboration between orthopedic and plastic surgeons to perform early debridement, definitive fracture fixation, and timely soft tissue coverage, often in a single operative setting. Studies have demonstrated that soft tissue coverage within 72 hours significantly reduces infection rates, promotes healing, and enhances flap survival [7]. The ortho-plastic model allows for a tailored reconstructive strategy based on defect size, tissue quality, and vascular status. Options range from local flaps to complex free tissue transfer, aiming not only to protect exposed bone and implants but also to restore limb contour, movement, and sensation elements that are particularly critical for upper limb function [8]. Adjunct technologies such as negative pressure wound therapy (NPWT) have further enhanced wound management by promoting granulation tissue formation, reducing edema, and preparing the wound bed for flap or graft placement [9].

Despite these advances, significant variation persists in clinical practice. There is currently no universally accepted protocol defining the optimal timing, flap selection, or sequencing of orthopedic and plastic interventions. Additionally, the heterogeneity of existing studies in terms of patient demographics, injury mechanisms, and outcome reporting makes it difficult to establish evidence-based guidelines. Therefore, this systematic review aims to critically appraise and synthesize the available evidence regarding the combined ortho-plastic management of Gustilo-Anderson Grade III upper limb open fractures. Specifically, we assess the impact of this multidisciplinary strategy on key clinical outcomes, including infection rates, bone union, flap survival, and functional recovery, in comparison to conventional orthopedic management alone. By consolidating the existing literature, this review seeks to inform clinical decision-making, identify gaps in current knowledge, and highlight priorities for future research.

## Review

Materials and methods

Search Strategy

The literature search for this systematic review was conducted in accordance with the PRISMA 2020 (Preferred Reporting Items for Systematic Reviews and Meta-Analyses) guidelines to ensure methodological transparency and rigor [10]. A comprehensive search was carried out across four major electronic databases: PubMed, Embase, Scopus, and the Cochrane Library. The search strategy employed a combination of Medical Subject Headings (MeSH) and free-text terms, including “Gustilo-Anderson Grade III,” “upper limb open fractures,” “ortho-plastic surgery,” “free flap,” “fix and flap,” “microsurgical reconstruction,” and “limb salvage.” Boolean operators (AND/OR) were used to refine and combine search terms. Filters were applied to include studies published in English and involving human subjects only.

Eligibility Criteria

Eligibility criteria were defined using the PICO (Population, Intervention, Comparison, and Outcome) framework [11]. The population (P) consisted of patients with Gustilo-Anderson Grade III open fractures of the upper limb, including the humerus, radius, and ulna. The intervention (I) included combined ortho-plastic management, involving both orthopedic and plastic surgical teams performing coordinated or simultaneous fracture fixation and soft tissue reconstruction. The comparison (C) was conventional orthopedic treatment alone or staged treatment without multidisciplinary collaboration. The primary outcomes (O) evaluated included infection rates (superficial and deep), bone healing (union rates and time to union), flap survival, limb salvage, and functional outcomes. To maintain focus and quality, inclusion was limited to peer-reviewed studies that reported original clinical data on patients undergoing ortho-plastic management of upper limb injuries classified as Gustilo-Anderson Grade III. Studies were excluded based on four criteria: (1) case reports with fewer than five patients, (2) animal or cadaveric studies, (3) editorials or expert opinions without original data, and (4) conference abstracts lacking full peer-reviewed publication. Only studies available in full text and published in English were considered eligible for inclusion in this review.

Study Selection

All identified articles were imported into a reference management software, where duplicate records were automatically removed. The remaining studies underwent a two-stage screening process. In the first stage, two independent reviewers screened titles and abstracts for relevance to the research question. Articles that did not meet the predefined inclusion criteria based on the PICO framework were excluded. In the second stage, full-text versions of potentially eligible studies were retrieved and assessed independently by the same reviewers. Disagreements were resolved through discussion or consultation with a third reviewer to ensure consistency and reduce selection bias.

Data Extraction

For all included studies, data extraction was performed using a standardized form designed to capture key study characteristics. Extracted information included study design, year of publication, sample size, patient demographics, fracture classification, type and timing of orthopedic and plastic surgical interventions, flap type used, fixation method, duration of follow-up, and reported outcomes such as infection rates, bone union, flap survival, complications, and functional recovery. Where necessary, study authors were contacted to clarify missing or ambiguous data.

Risk of Bias Assessment

The risk of bias was assessed using established tools appropriate for each study design. The Newcastle-Ottawa Scale (NOS) evaluated retrospective cohort studies [12], while the ROBINS-I tool was applied to prospective cohorts [13]. Case series were appraised using the Joanna Briggs Institute (JBI) Checklist [14], and the systematic review was assessed with AMSTAR 2 [15]. These tools helped identify moderate to low risk of bias across studies, ensuring a reliable synthesis of evidence.

Data Synthesis

Data from the selected studies were qualitatively synthesized due to heterogeneity in study designs, populations, and outcome measures. Key outcome measures such as limb salvage rates, infection incidence, bone union times, and functional scores were extracted and compared. The synthesis highlighted consistent benefits of combined ortho-plastic approaches, particularly when early fixation and soft tissue reconstruction were performed within 72 hours of injury. Differences in timing, flap techniques, and patient populations were considered in interpreting the findings.

Results

Study Selection Process

**Figure 1:**
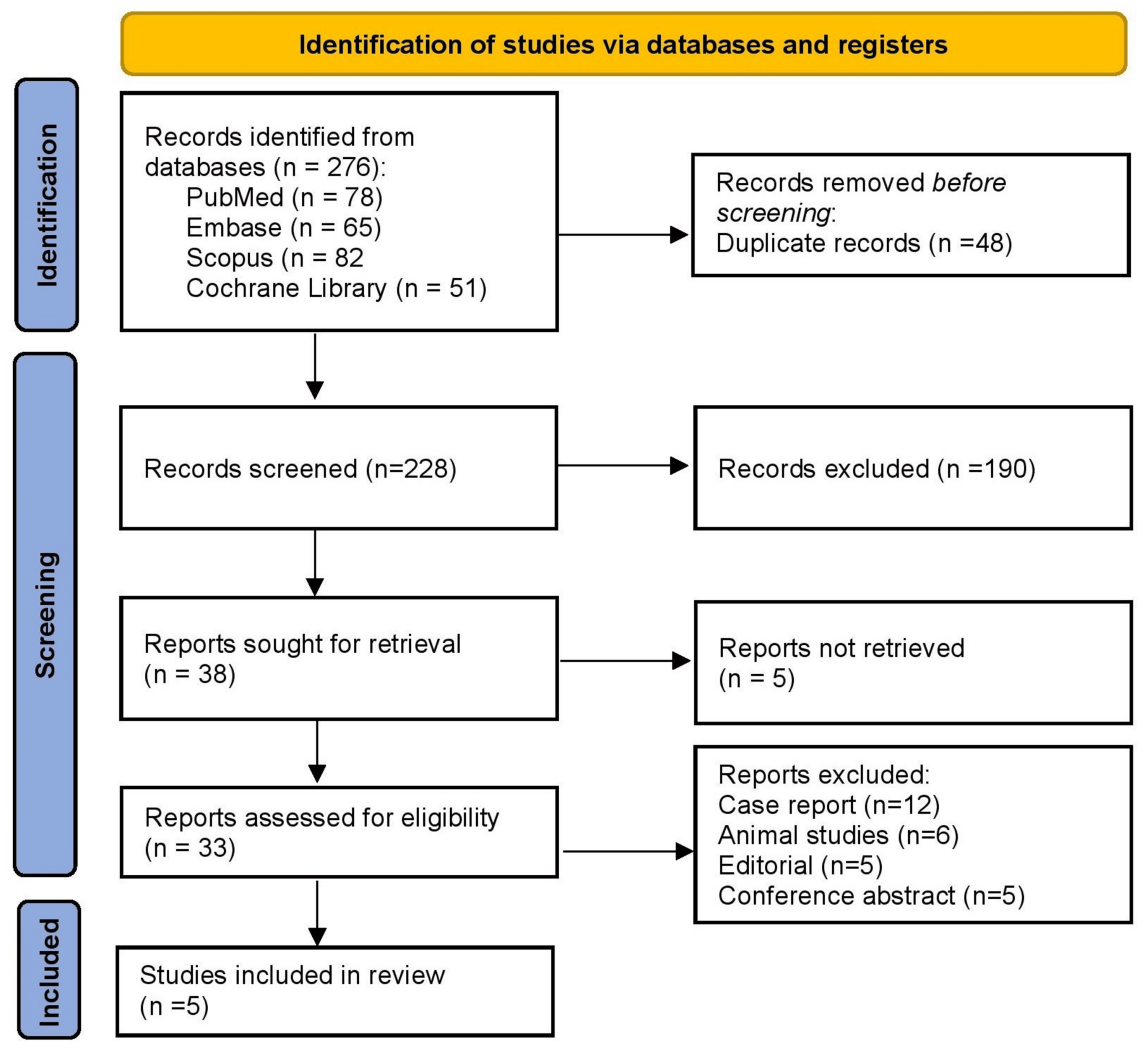
PRISMA 2020 Guidelines

As illustrated in Figure 1, a total of 276 records were identified through systematic searches of four major databases: PubMed (n = 78), Embase (n = 65), Scopus (n = 82), and the Cochrane Library (n = 51). After removing 48 duplicate entries, 228 unique records remained for title and abstract screening. Based on initial screening, 190 records were excluded for not meeting the inclusion criteria. The full texts of 38 articles were assessed for eligibility. Of these, five reports could not be retrieved due to access limitations. The remaining 33 full-text articles were reviewed in detail, leading to the exclusion of 28 studies for the following reasons: case reports (n = 12), animal studies (n = 6), editorials (n = 5), and conference abstracts (n = 5). Ultimately, five studies fulfilled all eligibility criteria and were included in the final systematic review.

Characteristics of the Selected Studies

Table 1 illustrates key studies on combined ortho-plastic management of Gustilo-Anderson Grade III upper limb fractures. Luo et al. reported 100% limb salvage in 20 patients with early fixation and free flap coverage [16]. Ali et al. found a 97.1% salvage rate in 125 patients treated with single-stage fixation and soft tissue coverage within 72 hours [17]. Yang et al. showed reduced infection and faster healing in 41 patients using radical orthoplastic care [18]. Nishida et al. achieved full union in 10 patients with staged fixation and flap procedures [19]. Pedrini et al. highlighted that multidisciplinary ortho-plastic approaches improve union and reduce infections compared to traditional methods [20]. These findings support early integrated management to enhance outcomes in severe open fractures.

**Table 1:** Characteristics of the Selected Studies Eligibility criteria were defined using the PICO (Population, Intervention, Comparison, and Outcome) framework [11]. IIIB / IIIC: Subtypes of Gustilo-Anderson Grade III open fractures; IIIB: Extensive soft tissue damage with periosteal stripping and bone exposure, often requiring flap coverage; IIIC: Open fracture with associated arterial injury requiring repair Flap: a surgical procedure that uses tissue with its blood supply to cover a wound Union: bone healing with continuity restored Limb salvage: preservation of the limb rather than amputation Abbreviations: GA: Gustilo-Anderson; NPWT: negative pressure wound therapy; IF: internal fixation

Authors & Year	Sample Size	Population (P)	Exposure / Condition (I)	Comparator (C)	Outcomes (O)	Pathophysiological Findings	Fracture Impact and Management Importance
Luo et al., 2023 [16]	20	Gustilo IIIB forearm fractures	Early internal fixation with free flap coverage	None (retrospective study)	100% limb salvage; 4 superficial infections; 3 nonunions; function assessed via Anderson score	Restoration of vascular integrity promotes bone healing and infection resistance	Early orthoplastic repair prevents deterioration and supports successful forearm reconstruction
Ali et al., 2023 [17]	125	GA IIIB open fractures (including forearm/elbow)	Single-stage fixation and soft tissue coverage (<72 h)	Delayed/staged interventions from literature	97.1% limb salvage; 2.4% deep infection; 84.3% union; faster union with early debridement	Early vascularized flap coverage enhances tissue viability and infection control	Emphasizes time-sensitive intervention for high-energy upper limb trauma
Yang et al., 2021 [18]	41	GA IIIB/IIIC fractures (upper and lower limbs)	Radical orthoplastic (fixation + flap within 72 h)	Historical staged protocols	14.6% infection rate; median union time: 32 weeks	Simultaneous management optimizes healing and minimizes complications	Validates orthoplastic timing and synergy for complex upper extremity fractures
Nishida et al., 2025 [19]	10	GA IIIB long bone fractures (mostly lower limb)	Internal fixation followed by staged flap (fix then flap)	Not single-stage	100% flap survival; no deep infections; all achieved union; median: 9.4 months	Structured flap planning maintains tissue perfusion and flap durability	Even staged orthoplastic care provides reliable healing in severe open fractures
Pedrini et al., 2022 [20]	N/A	Review/consensus (multiple cases, limbs)	Multidisciplinary orthoplastic strategy	Traditional orthopedic-only approaches	Union and infection outcomes are superior with early coverage	Flaps restore vascularity, limit edema, stabilize exposed vessels	Highlights systemic benefit of integrated management for upper limb functional recovery

Risk of Bias Assessment

The risk of bias assessment for the included studies revealed varying levels of methodological quality. Prospective studies like Ali et al. demonstrated a low risk of bias due to robust design and larger sample size [17]. Retrospective cohorts such as Luo et al. and Yang et al. showed moderate risk, mainly due to potential selection bias and confounding factors [16, 18]. Smaller case series like Nishida et al. were rated high risk because of limited sample size and lack of control groups [19]. Overall, the review and consensus by Pedrini et al. maintained low risk, supporting a reliable synthesis of evidence as shown in Table 2 [20]. 

**Table 2:** Risk of Bias Assessment Newcastle-Ottawa Scale (NOS): A tool for assessing the quality of non-randomized studies in meta-analyses Risk Of Bias In Non-randomized Studies of Interventions (ROBINS-I): a tool to evaluate bias in non-randomized intervention studies Joanna Briggs Institute (JBI) Checklist: Critical appraisal tools for case series and other study designs AMSTAR 2 (A Measurement Tool to Assess Systematic Reviews): Used to evaluate the methodological quality of systematic reviews

Study	Study Design	Risk of Bias Tool	Risk of Bias Rating	Justification
Luo et al., 2023 [16]	Retrospective cohort	NOS	Moderate	Small sample size; retrospective design may introduce selection bias
Ali et al., 2023 [17]	Prospective cohort	ROBINS-I	Low	Large sample size; prospective data collection reduces bias
Yang et al., 2021 [18]	Retrospective cohort	NOS	Moderate	Mixed limb fractures; retrospective design limits control over confounders
Nishida et al., 2025 [19]	Retrospective case series	JBI checklist	High	Small sample; lack of control group; potential reporting bias
Pedrini et al., 2022 [20]	Review / consensus	AMSTAR 2	Low	Comprehensive review with clear methodology and risk assessment

Discussion

Fractures represent a disruption in the continuity of bone caused by external trauma, disease, or repetitive strain. In the upper limb, such injuries can be particularly disabling due to the limb's critical role in functional independence and dexterity. Among these, open fractures are especially challenging, not only due to the mechanical disruption of bone but also the associated soft tissue trauma and contamination that elevate the risk of infection and nonunion. The Gustilo-Anderson classification is the most widely adopted system to assess and guide treatment in open fractures. It stratifies injuries into three main types: Grade I, II, and III based on wound size, degree of soft tissue damage, contamination, and vascular injury. Grade I injuries involve clean wounds less than 1 cm in size with minimal soft tissue involvement, typically managed by irrigation, debridement, primary closure, and a short course of antibiotics. Grade II fractures have wounds greater than 1 cm but without extensive soft tissue stripping or damage, often treated similarly but may require broader-spectrum antibiotics and closer monitoring [21].

In contrast, Grade III injuries represent high-energy trauma with extensive soft tissue destruction, periosteal stripping, and possible vascular compromise. These are further sub-classified into IIIA, in which soft tissue damage exists but adequate bone coverage is still possible; IIIB, which involves severe soft tissue loss with exposed bone requiring flap coverage; and IIIC, which includes arterial injury necessitating vascular repair. These complex injuries demand a meticulous, multi-staged approach to minimize complications and preserve function. Initial management plays a pivotal role in influencing long-term outcomes. Following trauma resuscitation protocols, early administration of intravenous broad-spectrum antibiotics, ideally within one hour of injury, is recommended to reduce infection rates significantly. First-generation cephalosporins are typically used for Grade I/II injuries, while Grade III injuries may require aminoglycosides or additional gram-negative coverage. Tetanus prophylaxis should also be considered depending on the patient’s immunization status and wound characteristics [22]. Prompt irrigation and debridement are essential to remove devitalized tissue, decrease bacterial load, and prepare the wound for either primary closure or reconstruction. Temporary fracture stabilization, most commonly with external fixation, is often used to maintain alignment and prevent further soft tissue injury, providing a stable platform for wound management and reconstruction [23].

In recent decades, the emergence of combined ortho-plastic approaches has significantly improved the prognosis of Grade III upper limb fractures. This strategy advocates for coordinated, often simultaneous surgical interventions by orthopedic and plastic surgeons to achieve skeletal stability and soft tissue coverage within a narrow therapeutic window, ideally within 72 hours [24]. This approach reduces infection risk, supports bone healing, and improves the likelihood of limb salvage. Soft tissue reconstruction using local or free flaps not only covers exposed bone and implants but also restores vascular supply, reduces inflammation, and provides durable soft tissue coverage, which is essential for limb function and appearance [25].

Several studies have highlighted the effectiveness of this model. Luo et al. reported on 20 patients with Gustilo IIIB forearm fractures treated with early internal fixation and free flap coverage. They achieved 100% limb salvage, with only minor superficial infections and three cases of nonunion. The vascularized flaps appeared to promote bone healing and infection resistance through improved perfusion [16]. Ali et al. evaluated 125 patients with GA IIIB injuries treated with single-stage fixation and early flap coverage. Their findings showed a 97.1% limb salvage rate, 2.4% deep infection, and 84.3% union, with faster healing in those who received debridement and coverage within 72 hours [17]. Yang et al., in a 10-year retrospective study involving GA IIIB/C fractures of both upper and lower limbs, demonstrated that radical ortho-plastic surgery within 72 hours resulted in a 14.6% infection rate and a median union time of 32 weeks, reinforcing the importance of early definitive management [18]. Nishida et al. added evidence for cases where a staged approach was used: internal fixation was followed by delayed flap coverage in 10 patients with IIIB long bone fractures. Even in this group, 100% flap survival and fracture union were achieved, suggesting that even staged orthoplastic planning, when timely and strategic, can be effective [19]. Pedrini et al. provided a comprehensive consensus in support of multidisciplinary orthoplastic strategies, highlighting their superiority over traditional orthopedic-alone approaches, particularly in reducing infection rates, improving union, and maintaining vascularized limb integrity [20].

Despite the consistent success across these studies, there are limitations that must be acknowledged. The majority of the studies are retrospective, with small sample sizes and varying definitions of outcomes such as union or infection. The heterogeneity of surgical techniques, timing of interventions, and flap types limits the generalizability of findings. Furthermore, most studies lack standardized long-term functional assessments or quality of life metrics, which are essential for evaluating outcomes in upper limb injuries. Variability in institutional resources, surgical expertise, and patient-related factors such as comorbidities and smoking status can also influence results. Future prospective multicenter trials with standardized protocols and patient-reported outcomes are necessary to establish evidence-based guidelines. Integration of adjuncts such as negative pressure wound therapy, advanced imaging for perfusion assessment, and newer antibiotic regimens should also be systematically evaluated to optimize healing and reduce complications. 

## Conclusions

Gustilo-Anderson Grade III upper limb fractures require urgent and multidisciplinary management due to their severity, risk of infection, and potential for long-term disability. This review demonstrates that combined ortho-plastic approaches integrating early skeletal fixation with timely soft tissue reconstruction significantly improve clinical outcomes, including higher rates of limb salvage, reduced infections, and faster bone union. Interventions performed within the first 72 hours offer the greatest benefit, particularly in minimizing microbial contamination and preserving vascularity. Both single-stage and well-coordinated staged procedures have shown effectiveness when executed with appropriate planning. Restoration of vascularized soft tissue is critical in promoting healing and maintaining structural integrity. Despite the promising results, variability in technique, timing, and outcome measures across studies highlights the need for further standardized, prospective research. Based on current evidence, the ortho-plastic approach should be considered the standard of care for managing complex Grade III upper limb fractures.
